# Comparison of clinical remission and survival between CLAG and FLAG induction chemotherapy in patients with refractory or relapsed acute myeloid leukemia: a prospective cohort study

**DOI:** 10.1007/s12094-017-1798-8

**Published:** 2017-11-27

**Authors:** Y. Bao, J. Zhao, Z.-Z. Li

**Affiliations:** 1 Department of Hematology, Xiangyang No. 1 People’s Hospital, Hubei University of Medicine, Xiangyang, China; 2 Department of Hematology, The Central Hospital of Wuhan, Tongji Medical College, Huazhong University of Science and Technology, Wuhan, China; 3 Department of Hematology, Taihe Hospital, Affiliated to Hubei University of Medicine, 32 Renmin South Road, Shiyan, 442099 China

**Keywords:** CLAG, FLAG, Induction chemotherapy, Clinical remission, Overall survival, Refractory or relapsed acute myeloid leukemia (R/R AML)

## Abstract

**Purpose:**

To compare the clinical remission and survival between CLAG and FLAG induction chemotherapy in treating patients with refractory or relapsed acute myeloid leukemia (R/R AML).

**Methods:**

103 R/R AML patients were consecutively enrolled in this prospective cohort study. 55 patients were treated by CLAG induction chemotherapy as follows: 5 mg/m^2^/day cladribine (days 1–5); 2 g/m^2^/day cytarabine (days 1–5) and 300 μg/day filgrastim (days 0–5). While 48 patients were treated by FLAG: 30 mg/m^2^/day fludarabine (days 1–5), 2 g/m^2^/day cytarabine (days 1–5), and 300 μg/day filgrastim (days 0–5).

**Results:**

CLAG induction chemotherapy achieved 61.7% complete remission rate (CR) and 78.7% overall remission rate (ORR), which was similar with FLAG chemotherapy which realized 48.7% CR and 69.2% ORR. No difference of overall survival (OS) was discovered between two groups either. Age cytarabine 60 years, secondary disease, poor risk stratification and BM blast ≥ 42.7% and second or higher salvage therapy were independent factors for worse prognosis. Subgroups analysis revealed that in patients with second or higher salvage therapy, CLAG seemed to achieve a higher CR than FLAG. And in patients with relapsed disease, poor risk stratification or CR at first induction, CLAG seemed to realize a prolonged OS compared to FLAG.

**Conclusion:**

CLAG was equally effective to FLAG induction chemotherapy in total R/R AML patients, while CLAG seemed to be a better option than FLAG in patients with relapsed disease, poor risk stratification, CR at first induction or second or higher salvage therapies.

**Electronic supplementary material:**

The online version of this article (doi:10.1007/s12094-017-1798-8) contains supplementary material, which is available to authorized users.

## Introduction

Acute myeloid leukemia (AML) is a cancer featured by infiltration of bone marrow, blood, and other tissues resulting from clonal, abnormally differentiated and occasionally poorly differentiated cells of hematopoietic system [1]. Although understanding in the pathology of AML has developed and a variety of therapies to AML have been used for many years, no response to induction therapy still happens in 20–40% and relapse after CR occurs in 50–70% of the patients, respectively, which are called refractory or relapsed AML (R/R AML) [2]. Moreover, the 1-year survival of those with first relapse is only 29% and it dropped remarkedly to 11% at 5-year [3]. Thus, it is necessary to explore effective therapies in treating R/R AML patients.

The common therapies for R/R AML patients include cytotoxic chemotherapy, targeted agents and immunotherapy [4]. As to chemotherapies, the salvage therapies based on cytarabine (Ara-C) plus other cytotoxic agents and cytokine are used widely in R/R AML disease. Plenty of studies have revealed that purine analogs as cytotoxic agents act as important parts in the salvage therapy for R/R AML through inhibiting ribonucleotide reductase (RNR) [4]. Among the purine analogs, fludarabine has been investigated for many years and it is usually combined with Ara-C and granulocyte colony-stimulating factor (G-CSF) (FLAG) as a salvage therapy in AML patients [5, 6]. It is reported that FLAG achieves CR ranging from 46 to 63% in R/R AML patients and the efficacy of fludarabine is on account of its triphosphate which inhibits RNR and elevates Ara-CTP in leukemic cells [7–9]. While another cytotoxic agent cladribine, as a new generation of purine analog, has been considered to be a substitute for fludarabine in the combination with Ara-C plus G-CSF (CLAG) as a treatment in R/R AML patients [10], which is not only due to its same mechanism as fludarabine, but also attribute to its additional effects that cladribine induces cells apoptotic process through changing the membrane potential of mitochondria, repressing DNA methyltransferase (DNMT) as well as consuming methyl donors and, thus, we hypothesized that CLAG might be more effective than FLAG [2, 11–13]. Based on the above advantages of CLAG, it has attracted the attention of Polish Adult Leukemia Group (PALG) and PALG has conducted a multicenter, open and non-controlled phase II clinical trial in R/R AML patients in which CLAG achieves CR of 50% and 1-year OS of 42% [14]. In Chinese population, CLAG has been disclosed to achieve CR up to 78.8% in R/R AML patients as well as a satisfactory median OS of 419 days and, thus, CLAG has been proposed as the first-line therapy in R/R AML patients due to its good performance [15, 16]. However, the difference of outcomes between CLAG and FLAG is still unclear; thus, we conducted this study to investigate the difference of clinical remission and survival between the two treatments as well as predictive factors which may affect the outcomes in R/R AML patients.

## Materials and methods

### Patients

103 R/R AML patients between Jan 2013 and Dec 2015 were consecutively enrolled in this prospective cohort study. The inclusion criteria were as follows: (1) diagnosed as AML according to the classification of morphology, immunology, cytogenetics, molecular biology (MICM), and categorized as relapsed or refractory AML; (2) bone marrow (BM) blasts above 10%. (3) About to receive urine analogue combination chemotherapy as induction (CLAG treatment or FLAG treatment). The exclusions were: (1) previously treated by urine analog-based therapy. (2) Severe heart dysfunction, severe arrhythmia, severe lung dysfunction, severe hepatic dysfunction or renal dysfunction; (3) pregnancy, lactation or planed for pregnancy. Patient was included in the analysis only once (the first salvage treatment after enrollment) if multiple lines of salvage therapies were undergone after the recruitment. This study was approved by the Ethics Committee of Taihe Hospital Affiliated to Hubei University of Medicine; each patient signed the informed consents.

### Treatment

This study did not intervene the treatments of patients, and all patients were treated by CLAG or FLAG induction chemotherapy by disease conditions, clinical experience and patients’ decisions. CLAG treatment was as follows: 5 mg/m^2^/day cladribine (days 1–5); 2 g/m^2^/day cytarabine (days 1–5) and 300 μg/day filgrastim (days 0–5). FLAG treatment was as follows: 30 mg/m^2^/day fludarabine (days 1–5), 2 g/m^2^/day cytarabine (days 1–5), and 300 μg/day filgrastim (days 0–5). In CLAG treatment group, 5 patients were combined with previous mitoxantrone treatment at 10 mg/m^2^/day for 3 days. In FLAG treatment group, 6 patients were combined with previous idarubicin treatment at 10 mg/m^2^/day for 3 days.

### Baseline data collection

Baseline patients’ characteristics were recorded including: induction treatment regimen, age, gender, disease status, de novo or secondary, poor risk stratification, Eastern Cooperative Oncology Group (ECOG) performance score, BM blasts, Complete remission (CR) status at first induction, previous allogeneic hematopoietic stem-cell transplantation (allo-HSCT) and line of salvage therapy.

### Definitions and assessments

The R/R AML was defined as: Refractory AML—(1) patients do not achieve CR after two courses of induction chemotherapy by standard protocol; (2) patients relapse within 6 months after first CR; (3) patients relapse at 6 months or above after first CR and fail by the subsequent induction chemotherapy; (4) patients relapse more than 2 times; (5) extra-medullary infiltration of leukemia. Relapsed AML-leukemic cells reappeared in peripheral blood or the percentage of bone marrow blasts above 10%. Secondary AML was defined as therapy-related AML or other hematological malignancy-related AML. Risk stratification in our study was assessed according to the NCCN Guidelines (NCCN Guidelines 2013 Acute Myeloid Leukemia) based on patients’ cytogenetics and molecular abnormalities. The detailed classification criteria were as follows: (1) Good risk: cytogenetics including inv(16), t(16;16), t(8;21), t(15;17), or normal cytogenetics with NPM1 mutation in the absence of FLT3-ITD or isolated biallelic CEBPA mutation; (2) standard risk: normal cytogenetics +8 alone, t(9;11) or other non-defined, or molecular abnormalities including t(8;21), inv(16) or t(16;16) with c-KIT mutation; (3) Poor risk: Complex (≥ 3 clonal chromosomal abnormalities), -5, 5q-, -7, 7q-, 11q23—non t(9;11), inv(3), t(3;3), t(6;9) or t(9;22), or normal cytogenetics with FLT3-ITD mutation. CR status at first induction was defined as whether CR was achieved at first induction therapy after initial diagnosis of AML.

CR was evaluated as BM with at least 20% cellularity and BM blasts below 5% at steady state after chemotherapy, without cytological evidence of leukemia, no transfusion requirement, and leukocyte count above 1 × 10^9^/l as well as platelet count above 100 × 10^9^/l. Partial remission (PR) was defined as either BM blasts 5–25%, or a 50% or better decrease in BM blasts, or BM blasts < 5% but with Auer rods’ presence. Overall remission rate (ORR) was defined as patients with CR and PR. Achievement of allo-HSCT was also assessed as an endpoint. Overall survival (OS) was calculated from the date of treatment to the time of death for any cause.

### Follow-ups

The median follow-up duration was 9 months (range 0.5–45 months), and the last follow-up date was Dec 2016.

### Statistics

Data were analyzed using SPSS 21.0 software (IBM, USA) and OFFICE 2010 software (Microsoft, USA). Comparison between two group was determined by *t* test, Wilcoxon rank sum test or Chi square test; Kaplan–Meier (K–M) curves were performed to analyze the OS and log-rank test was used to determine the difference of OS between/among groups; univariate and multivariate logistic regression was performed to analyze the factors affecting CR achievement; univariate and multivariate Cox’s proportional hazards regression was performed to analyze the factors affecting OS. In addition, patients were divided into subgroups and the efficacy of CLAG and FLAG treatment was also compared in subgroups. *p* < 0.05 was considered significant.

## Results

### Baseline characteristics

55 patients, 31 males and 24 females, were enrolled in CLAG group with mean age of 51.0 ± 17.7 years in which 32 (58%) were relapsed disease and 23 (48%) were refractory disease (Table 1). 48 patients, 27 males and 21 females, were enrolled in FLAG group with mean age of 48.8 ± 17.5 years in which 29 (60%) were relapsed and 19 (40%) were refractory disease. No difference of age (*p* = 0.523), gender (*p* = 0.991) and disease status (*p* = 0.658) was observed between two groups. 44 (80%) and 11 (20%) cases in CLAG group were de novo and secondary AML disease, while the numbers in FLAG group were 37 (77%) and 11(23%), respectively (*p* = 0.719). Based on cytogenetics and molecular abnormalities, 6 (11%), 30 (54), 17 (31%) patients in CLAG treatment were classified into good, standard and poor risk with additionally 2 (4%) patients were not evaluated for risk stratification, in the meanwhile, 9 (19%), 21 (44%), 16 (33%) and 1 (2%) patients in FLAG treatment were categorized in good, standard, poor and unknown risk (*p* = 0.586). All cases were also evaluated according to ECOG performance, which showed that 18 (33%), 33 (60%) and 4 (7%) cases in CLAG group were with ECOG performance score 0, 1 and 2; meanwhile, 13 (27%), 30 (63%) and 5 (10%) cases in FLAG group were with ECOG performance score 0, 1 and 2 (*p* = 0.745). BM blast at baseline in CLAG and FLAG was 43.2 (25.1–61.8) and 41.6 (29.5–64.8), respectively (*p* = 0.761). Patients with CR at first induction were 28 (51%) and 20 (42%) in CLAG and FLAG group, respectively (*p* = 0.348). In addition, 11 (20%) cases in CLAG and 10 (21%) in FLAG had undergone allo-HSCT previously (*p* = 0.917); moreover, 14 (25%) cases in CLAG and 10 (21%) cases in FLAG were at second or higher salvage therapy (*p* = 0.580).

**Table 1:** Baseline characteristics

Parameters	CLAG treatment (*N* = 55)	FLAG treatment (*N* = 48)	*p* value
Age (years)	51.0 ± 17.7	48.8 ± 17.5	0.523
Gender (male/female)	31/24	27/21	0.991
Disease status			0.658
Relapsed (*n*/%)	32 (58)	29 (60)	
Refractory (*n*/%)	23 (42)	19 (40)	
De novo or secondary			0.719
De novo (c %)	44 (80)	37 (77)	
Secondary (*n*/%)	11 (20)	11 (23)	
Risk stratification			0.586
Good (*n*/%)	6 (11)	9 (19)	
Standard (*n*/%)	30 (54)	21 (44)	
Poor (*n*/%)	17 (31)	16 (33)	
Unknown (*n*/%)	2 (4)	1 (2)	
ECOG performance			0.745
0 (*n*/%)	18 (33)	13 (27)	
1 (*n*/%)	33 (60)	30 (63)	
2 (*n*/%)	4 (7)	5 (10)	
BM blast (%)	43.2 (25.1–61.8)	41.6 (29.5–64.8)	0.761
CR at first induction			0.348
Yes (*n*/%)	28 (51)	20 (42)	
No (*n*/%)	27 (49)	28 (58)	
Previous allo-HSCT (*n*/%)			0.917
Yes (*n*/%)	11 (20)	10 (21)	
No (*n*/%)	44 (80)	38 (79)	
Lines of salvage therapy			0.580
First salvage therapy (*n*/%)	41 (75)	38 (86)	
Second or higher salvage therapy (*n*/%)	14 (25)	10 (21)	

Data were presented as mean value and standard deviation, median value and 1/4–3/4 quarters, or count (percentage). Comparison was determined by t test, Wilcoxon rank sum test or Chi square test. *p* < 0.05 was considered significant

*ECOG* Eastern Cooperative Oncology Group, *BM* bone marrow, *CR* complete remission, *allo*-*HSCT* allogeneic hematopoietic stem-cell transplantation

### Clinical efficacy by CLAG and FLAG treatments

**Fig. 1:**
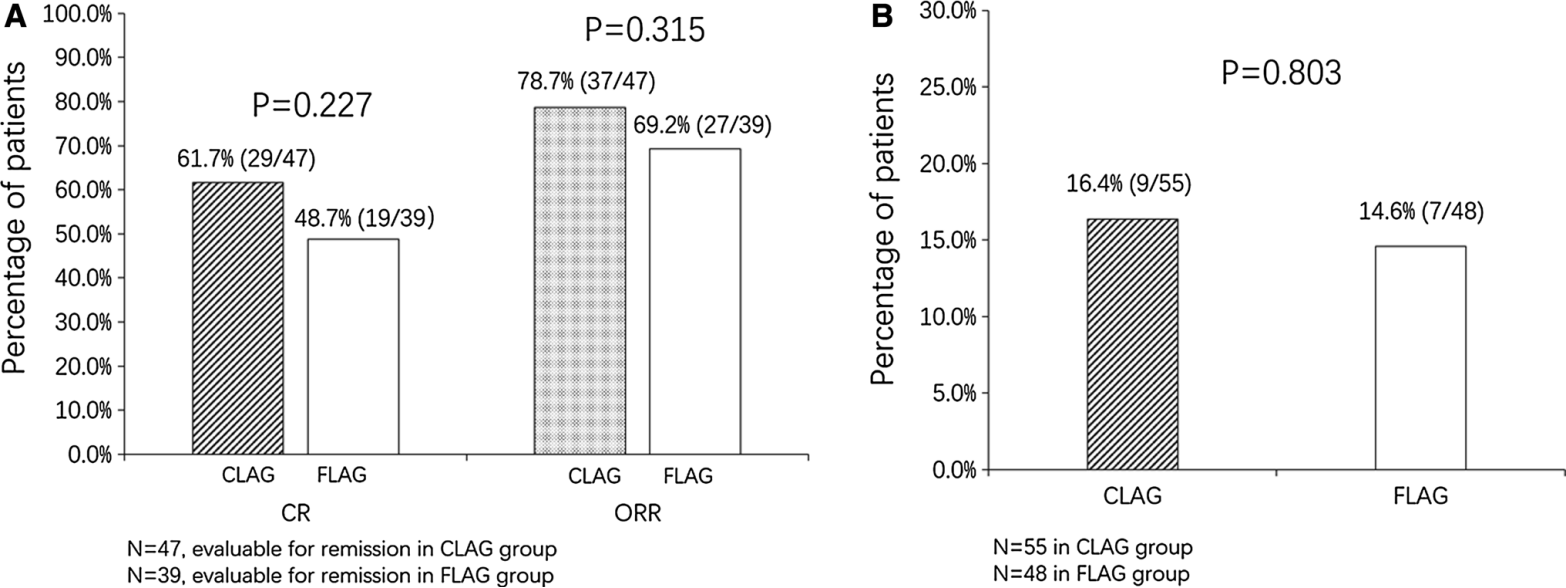
Clinical efficacy after CLAG and FLAG therapies. **a** 47/55 patients in CLAG group were evaluable for remission in which 61.7% achieved CR and 78.7% achieved ORR; 39/48 patients in FLAG group were evaluable for remission in which 48.7% achieved CR and 69.2% achieved ORR. No difference of CR or ORR between CLAG and FLAG was observed. **b** Achievement of allo-HSCT after CR. 16.4% (9/55) and 14.6% (7/48) in CLAG and FLAG groups received allo-HSCT, respectively, with no difference between two groups. Comparison between two groups was determined by Chi square test. *p* < 0.05 was considered significant

As shown in Fig. 1, 47 and 39 patients were evaluable for remission in CLAG and FLAG group, respectively. 61.7% (29/47) patients in CLAG group and 48.7% (19/39) patients in FLAG group achieved CR (*p* = 0.227) (Fig. 1a); meanwhile, 78.7% (37/47) cases in CLAG group and 69.2% (37/47) in FLAG group obtained ORR (*p* = 0.315) (Fig. 1a). Both CR and ORR presented no difference between two groups. In addition, no difference was observed in allo-HSCT achievement after treatments between CLAG and FLAG (16.4 vs. 14.6%, *p* = 0.803) (Fig. 1b).

### Overall survival

**Fig. 2:**
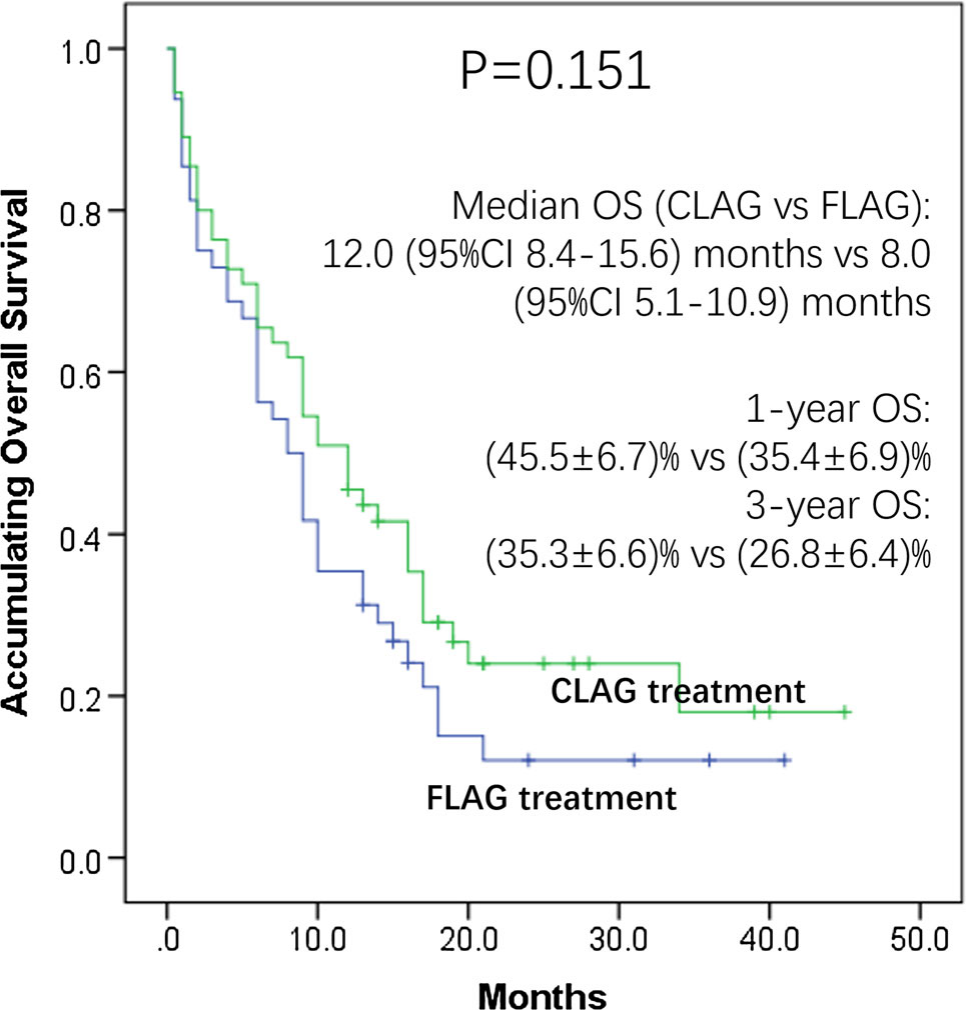
Accumulating OS by CLAG and FLAG treatments. K–M curve analysis of accumulating OS. CLAG therapy achieved median OS of 12.0 (95% CI 8.4–15.6) months and FLAG achieved median OS of 8.0 (95% CI 5.1-10.9) months. The 1-year OS of CLAG and FLAG group was (45.5 ± 6.7) % and (35.4 ± 6.9) %, and 3-year OS was (35.3 ± 6.6) % and (26.8 ± 6.4) % respectively. No difference of OS was observed. Comparison of OS between groups was determined by K–M curves and log-rank test. *p* < 0.05 was considered significant

K–M curve and log-rank test were conducted to analyze the accumulating OS (Fig. 2), which revealed that no difference of OS was observed between CLAG and FLAG treatments (*p* = 0.151). Median OS of CLAG and FLAG was 12.0 (95% CI 8.4–15.6) months and 8.0 (95% CI 5.1–10.9) months. CLAG attained 1-year OS of (45.5 ± 6.7) % and 3-year OS of (35.3 ± 6.6)%, while FLAG achieved 1-year OS of (35.4 ± 6.9)% and 3-year OS of (26.8 ± 6.4)%.

### Analysis of factors affecting CR

Factors affecting CR were assessed by univariate logistic regression (Table 2). Age ≥ 60 years (*p* = 0.001), poor risk stratification (*p* = 0.003), BM blast ≥ 42.7% (*p* = 0.030), second or higher salvage therapy (*p* = 0.049) were associated with less possibility of achieving CR. While CR at first induction was correlated with the presence of CR (*p* = 0.024). All factors were subsequently analyzed via multivariate model, which disclosed that age ≥ 60 years (*p* = 0.016), poor risk stratification (*p* = 0.029) and BM blast ≥ 42.7% (*p* = 0.022) were independent predictive factors for the absence of CR.

**Table 2:** Univariate and multivariate logistic regression analysis of factors affecting CR

Parameters	Univariate logistic regression				Multivariate logistic regression			
	*p* value	OR	95% CI		*p* value	OR	95% CI	
			Lower	Higher			Lower	Higher
CLAG treatment (vs. FLAG treatment)	0.315	1.551	0.659	3.653	0.223	1.988	0.658	6.006
Age ≥ 60 years	**0.001**	0.213	0.084	0.538	**0.016**	0.224	0.066	0.760
Male (vs. female)	0.621	0.806	0.344	1.892	0.849	0.891	0.272	2.921
Relapsed disease (vs. refractory)	0.349	1.513	0.636	3.597	0.502	1.577	0.417	5.968
Secondary disease (vs. de novo)	0.335	0.583	0.195	1.744	0.753	1.263	0.294	5.415
Higher risk stratification	**0.003**	0.321	0.152	0.682	**0.029**	0.363	0.146	0.903
Higher ECOG performance	0.246	1.645	0.710	3.814	0.236	2.000	0.635	6.300
BM blast ≥ 42.7%	**0.030**	0.380	0.158	0.913	**0.022**	0.239	0.071	0.810
CR at first induction	**0.024**	2.786	1.143	6.787	0.807	1.198	0.282	5.083
Previous allo-HSCT	0.355	1.681	0.559	5.055	0.805	1.232	0.235	6.445
Second or higher salvage therapy (vs. first)	**0.049**	0.350	0.123	0.993	0.493	0.610	0.148	2.507

Data were presented as *p* value, OR (odds ratio) and 95% CI. Factors affecting CR achievement were determined by univariate logistic regression analysis and multivariate logistic regression analysis. *p* value < 0.05 was considered significant. Risk stratification was scored as 1-good; 2-standard; 3-poor. The analysis was based on this definition

Bold values are data with* p* value < 0.05, which were considered significant

*ECOG* Eastern Cooperative Oncology Group, *BM* bone marrow, *CR* complete remission, *allo*-*HSCT* allogeneic hematopoietic stem-cell transplantation

### Analysis of factors affecting ORR

As to factors affecting ORR, univariate logistic regression in Table 3 displayed that age ≥ 60 years was associated with worse ORR (*p* = 0.002). All factors were analyzed through the following multivariate model, which presented age ≥ 60 years was an independent predictive factor predicting less possibility to achieve ORR (*p* = 0.009).

**Table 3:** Univariate and multivariate logistic regression analysis of factors affecting ORR

Parameters	Univariate logistic regression				Multivariate logistic regression			
	*p* value	OR	95% CI		*p* value	OR	95% CI	
			Lower	Higher			Lower	Higher
CLAG treatment (vs. FLAG treatment)	0.317	1.644	0.620	4.359	0.199	2.191	0.661	7.259
Age ≥ 60 years	**0.002**	0.197	0.069	0.560	**0.009**	0.181	0.050	0.653
Male (vs. female)	0.704	1.207	0.458	3.183	0.136	3.060	0.704	13.305
Relapsed disease (vs. refractory)	0.305	1.667	0.627	4.427	0.306	2.128	0.501	9.038
Secondary disease (vs. de novo)	0.072	0.351	0.112	1.097	0.689	0.733	0.161	3.344
Higher risk stratification	0.057	0.454	0.201	1.023	0.079	0.366	0.119	1.123
Higher ECOG performance	0.662	0.808	0.310	2.102	0.386	0.546	0.139	2.144
BM blast ≥ 42.7%	0.390	0.650	0.244	1.735	0.423	0.578	0.151	2.207
CR at first induction	0.069	2.667	0.925	7.685	0.545	0.624	0.135	2.878
Previous allo-HSCT	0.161	3.061	0.640	14.632	0.212	3.718	0.473	29.228
Second or higher salvage therapy (vs. first)	0.097	0.404	0.138	1.179	0.661	0.716	0.162	3.178

Data were presented as *p* value, OR (odds ratio) and 95% CI. Factors affecting ORR achievement were determined by univariate logistic regression analysis and multivariate logistic regression analysis. *p* value < 0.05 was considered significant. Risk stratification was scored as 1-good; 2-standard; 3-poor. The analysis was based on this definition

Bold values are data with* p* value < 0.05, which were considered significant

*ECOG* Eastern Cooperative Oncology Group, *BM* bone marrow, *ORR* overall remission rate, *CR* complete remission, *allo*-*HSCT* allogeneic hematopoietic stem-cell transplantation

### Analysis of factors affecting OS

As displayed in Table 4, age ≥ 60 years (*p* = 0.005), secondary disease (*p* = 0.005), poor risk stratification (*p* = 0.004), BM blast ≥ 42.1% (*p* = 0.021) and second or higher salvage therapy (*p* = 0.003) were all correlated with worse OS. Subsequently, all factors were evaluated by multivariate Cox’s regression which illustrated that secondary disease (*p* = 0.042), BM blast ≥ 42.1% (*p* = 0.001) and second or higher salvage therapy (*p* = 0.009) were independent predictive factors predicting poor OS.

**Table 4:** Univariate and multivariate Cox’s proportional hazards regression analysis of factors affecting OS

Parameters	Univariate Cox regression				Multivariate Cox regression			
	*p* value	HR	95% CI		*p* value	HR	95% CI	
			Lower	Higher			Lower	Higher
CLAG treatment (vs. FLAG treatment)	0.166	0.734	0.474	1.137	0.135	0.700	0.439	1.117
Age ≥ 60 years	**0.005**	1.870	1.206	2.901	0.064	1.567	0.974	2.52
Male (vs. female)	0.279	1.278	0.820	1.993	0.612	1.156	0.661	2.021
Relapsed disease (vs. refractory)	0.164	0.727	0.465	1.139	0.891	0.959	0.527	1.746
Secondary disease (vs. de novo)	**0.005**	2.105	1.255	3.532	**0.042**	1.977	1.024	3.819
Risk stratification	**0.004**	1.634	1.167	2.289	0.068	1.424	0.975	2.079
ECOG performance	0.083	1.441	0.954	2.175	0.243	1.356	0.813	2.262
BM blast ≥ 42.1%	**0.021**	1.698	1.084	2.660	**0.001**	2.466	1.455	4.180
CR at first induction	0.078	0.670	0.429	1.046	0.151	0.629	0.334	1.185
Previous allo-HSCT	0.137	0.644	0.361	1.150	0.631	1.140	0.668	1.947
Second or higher salvage therapy (vs. first)	**0.003**	2.173	1.312	3.597	**0.009**	2.313	1.238	4.319

Data were presented as *p* value, HR (Hazards ratio) and 95% CI. Significance was determined by univariate and multivariate Cox’s proportional hazards regression analysis. *p* value < 0.05 was considered significant. Risk stratification was scored as 1-good; 2-standard; 3-poor. The analysis was based on this definition

Bold values are data with* p* value < 0.05, which were considered significant

*ECOG* Eastern Cooperative Oncology Group, *BM* bone marrow, *CR* complete remission, *allo*-*HSCT* allogeneic hematopoietic stem-cell transplantation

### Subgroup analysis with CR

To further explore the difference of efficacy between CLAG and FLAG, we divided all participants into different subgroups by baseline characteristics. As presented in Table 5, in patients with second or higher salvage therapy, CLAG achieved numerically higher CR compared to FLAG but presented no significant difference (*p* = 0.085). No difference of CR between CLAG and FLAG was discovered in other subgroups.

**Table 5:** Subgroups analysis of CR by CLAG and FLAG treatment

Parameters	CR in CLAG group	CR in FLAG group	*p* value
Total (*s*/%)	29/47 (62)	19/39 (49)	0.227
Age ≥ 60 years			
Yes (*n*/*N*/%)	7/18 (39)	4/16 (25)	0.388
No (*n*/*N*/%)	22/29 (76)	15/23 (65)	0.400
Gender			
Male (*n*/*N*/%)	15/25 (60)	9/21 (43)	0.246
Female (*n*/*N*/%)	14/22 (64)	10/18 (56)	0.604
Disease status			
Relapsed (*n*/*N*/%)	19/27 (70)	12/24 (50)	0.137
Refractory (*n*/*N*/%)	10/20 (50)	7/15 (47)	0.845
De novo or secondary			
De novo (*n*/*N*/%)	26/39 (67)	15/31 (48)	0.123
Secondary (*n*/*N*/%)	3/8 (38)	4/8 (50)	0.614
Risk stratification			
Good (*n*/*N*/%)	5/6 (83)	6/9 (67)	0.475
Standard (*n*/*N*/%)	19/26 (73)	11/19 (58)	0.286
Poor (*n*/*N*/%)	4/13 (31)	2/10 (20)	0.560
Unknown (*n*/*N*/%)	1/2 (50)	0/1 (0)	0.386
ECOG performance			
0 (*n*/*N*/%)	10/17 (59)	5/12 (42)	0.362
1 (*n*/*N*/%)	18/29 (62)	13/26 (50)	0.368
2 (*n*/*N*/%)	1/1 (100)	1/1 (100)	1.000
BM blast ≥ 42.1%			
Yes (*n*/*N*/%)	11/22 (50)	9/22 (41)	0.545
No (*n*/*N*/%)	18/25 (72)	10/17 (59)	0.374
CR at first induction			
Yes (*n*/*N*/%)	17/23 (74)	9/15 (60)	0.367
No (*n*/*N*/%)	12/24 (50)	10/24 (42)	0.562
Previous allo-HSCT			
Yes (*n*/*N*/%)	6/9 (67)	5/8 (63)	0.858
No (*n*/*N*/%)	23/38 (61)	14/31 (45)	0.203
Lines of salvage therapy			
First salvage therapy (*n*/*N*/%)	23/35 (66)	18/31 (58)	0.523
Second or higher salvage therapy (*n*/*N*/%)	6/12 (50)	1/8 (13)	0.085

There were totally 86 patients evaluable for remission (47 in CLAG group and 39 in FLAG group). Data were presented as count (percentage). Comparison was determined by Chi square test. *p* < 0.05 was considered significant

*ECOG* Eastern Cooperative Oncology Group, *BM* bone marrow, *CR* complete remission, *allo*-*HSCT* allogeneic hematopoietic stem-cell transplantation

### Subgroup analysis with OS

**Fig. 3:**
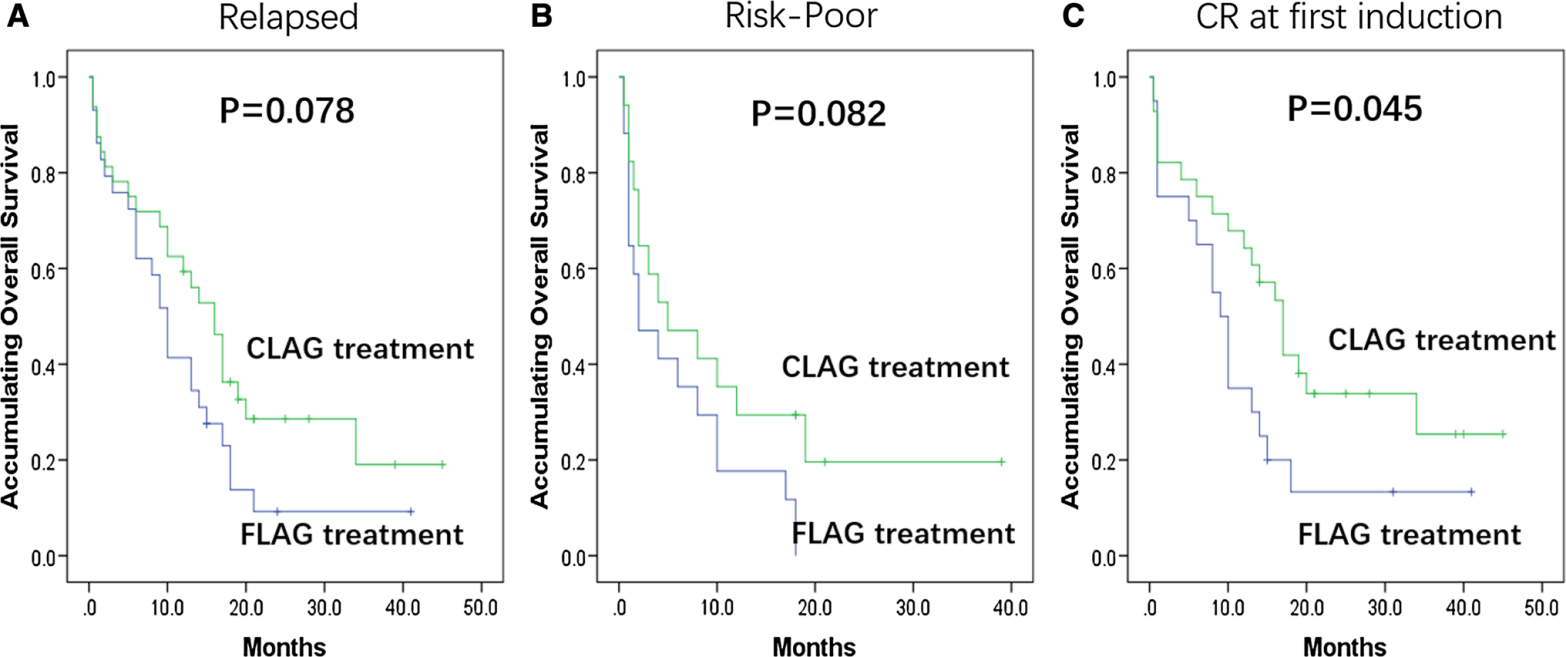
Accumulating OS between CLAG and FLAG treatments in subgroups divided by baseline characteristics (Data with *p* value < 0.1). Subgroup analysis of accumulating OS revealed that in patients with CR at first induction, CLAG achieved longer OS than FLAG (**c**). In patients with relapsed AML (**a**) and poor risk stratification (**b**), CLAG obtained numerically better OS compared to FLAG but no significant difference was found. Comparison of OS between groups was determined by K–M curves and log-rank test. *p* < 0.05 was considered significant

As to subgroups analysis on OS, CLAG achieved longer OS than FLAG in patients with CR at first induction (Fig. 3c, *p* = 0.045). In addition, in patients with relapsed disease (Fig. 3a, *p* = 0.078) and poor risk stratification (Fig. 3b, *p* = 0.082), CLAG attained numerically prolonged OS compared with FLAG but no significant difference was observed. No other difference of OS between CLAG and FLAG was found in subgroups divided by baseline characteristics (Supplemental Figure S1).

## Discussion

In our study, we evaluated difference of efficacy and survival between CLAG and FLAG in R/R AML patients and the two therapies achieved following outcomes: (1) CLAG and FLAG realized CR rates of 61.7 and 48.7% with median OS of 12.0 (95% CI 8.4–15.6) months and 8.0 (95% CI 5.1–10.9) months, respectively. There was no difference of CR and OS between two therapies; (2) age ≥ 60 years, poor risk stratification and BM blast ≥ 42.7% were verified to be independent predictive factors for unsatisfactory CR; meanwhile, secondary disease, BM blast ≥ 42.1% and second or higher salvage therapy were independent predictive factors predicting worse OS; (3) subgroups analysis was performed to further evaluate difference of outcomes between two therapies. Although CLAG and FLAG presented no difference of clinical remission and survival profile in total patients, CLAG therapy appeared to achieve better CR rates in patients with second or higher salvage therapy as well as prolonged OS in patients with relapsed disease, poor risk stratification or CR at first induction compared to FLAG.

Salvage therapies based on Ara-C plus G-CSF and purine analogs are widely used in R/R AML patients. Ara-C, as the backbone of these chemotherapies, is known for its cytotoxic effect in leukemic blasts which attributes to its active metabolites Ara-C-5′ triphosphate (Ara-CTP). Ara-CTP is the inhibitory factor of DNA polymerase which terminates the extension of DNA stands when binds to DNA, and then induces the apoptosis [11]. G-CSF as a cytokine has a popular application in hematological diseases such as myelodysplastic syndrome (MDS), AML, and chronic neutrophilic leukemia (CNL) [17, 18]. As to R/R AML, G-CSF not only mobilizes hematopoietic precursor cells but also promotes the differentiation of myeloid cells [18]. Purine analog which is represented for fludarabine was reported to be effective in R/R AML patients for years [4]. Purine analog increases the cellular uptake as well as accumulation of Ara-CTP in leukemic blasts which is responsible for cytotoxic effect. Moreover, fludarabine is demonstrated that it inhibits ribonucleotide reductase (RNR) and, thus, affects the cells proliferation. When fludarabine binds to DNA or RNA, it triggered the process of cells apoptosis [9]. Cladribine possesses the same mechanisms as fludarabine; in addition, it is able to change membrane potential of mitochondria which induces the cytochrome C and apoptosis inducing factor entering cytoplasm and, thus, leads to apoptosis [2, 11, 12]. On the other hand, cladribine represses DNMT and consumes methyl donors which is known as demethylation contributing to cell death [2, 11–13]. Other than above mechanisms, cladribine accumulates in some cells and consequently induces the programmed cell death [12]. All these illustrate the mechanisms of FLAG and CLAG therapy in R/R AML patients.

The efficacy of CLAG or FLAG in R/R AML patients has been verified. A comparison of efficacy between CLAG and another regimen including mitoxantrone, etoposide and Ara-C (MEC) in R/R AML patients has been performed by Samantha L. Price et al. in a retrospective study, and CLAG achieves higher CR than MEC therapy [19]. Wang Tao et al. compare the modified FLAG and another chemotherapy which consists of aclarubicin, Ara-C and G-SCF (CAG) in 61 R/R AML patients and, consequently, they obtain CR of 43% in modified FLAG group which is increased compared to CAG (CR of 21%) [3]. Although these studies disclose the satisfactory CR by CLAG or FLAG treatment for R/R AML, direct comparison of CR between CLAG and FLAG is still needed. A retrospective study based on cladribine-containing chemotherapy and fludarabine-containing chemotherapy has been conducted by Hyunkyung Park et al. to compare the efficacy of the two chemotherapies in R/R AML patients. Cladribine group in their study achieves a CR of 62.7% and fludarabine group achieves CR of 61.4%, which presents no difference between the two therapies [20]. In accordance with the previous study, we found that CLAG achieved CR of 61.7% and FLAG achieved 48.7% in R/R AML patients, respectively, and no significant difference was observed, indicating that CLAG was equally effective with FLAG in total R/R AML patients. This result might be on account of that: (1) fludarabine and cladribine both play roles in inducing cells apoptosis through inhibiting RNR and DNA stands which leading to a suppression of leukemia blasts [10, 13]; (2) Ara-C forms Ara-CTP which possesses cytotoxic effect and the produce and accumulation of Ara-CTP is greatly elevated by purine analogs including fludarabine and cladribine [4].

Survival profiles of CLAG or FLAG were also reported by several studies. A retrospective study comparing survival between CLAG and MEC for R/R AML shows numerically longer OS in CLAG group with the median OS of 7.3 months compared with MEC group (median OS: 4.5 months) [19]. A retrospective study evaluated the outcomes of FLAG and another chemotherapy using clofarabine, Ara-C and G-CSF (GCLAC) in R/R AML patients, with an additional therapy using fludarabine plus Ara-C without G-SCF (FA) serving as the control group. Their results that FLAG achieves median OS of 3.8 months which is shorter than GCLAC (8.8 months) demonstrates that FLAG may be an unfavorable choice for R/R AML patients compared to GCLAC. Interestingly, they have more refractory patients in GCLAC group than FLAG (36 vs. 15%) and, thus, the superiority of GCLAC may attribute to the higher sensitivity to clofarabine of refractory cases [21]. As to direct comparison of survival between CLAG and FLAG, a study performed by Hyunkyung Park et al. displays no difference of OS and recurrence-free survival (RFS) between CLAG and FLAG groups [20]. In line with the previous study, we found CLAG and FLAG achieved median OS of 12.0 (95% CI 8.4–15.6) months and 8.0 (95% CI 5.1–10.9) months, respectively, while no difference of OS was observed, and this satisfactory OS might be on account of the fact that: (1) the synergistic effect of purine analogs and Ara-C leads to a strong anti-leukemia effect through cytotoxic effects [22]; (2) for long-term survival, G-CSF improves the hematopoietic activity by promoting the differentiation of myeloid cells [18].

As to the factors affecting outcomes of CLAG and FLAG, the retrospective study conducted by Hyunkyung Park et al. verifies that age ≥ 60 years, poor cytogenetic risk status and larger number of prior induction therapies are predictive factors for unsatisfactory CR. In terms of survival, secondary AML as well as shorter first CR duration is independent factors predicting unfavorable OS, while CLAG regimen and allo-HSCT after CR are independent factors predicting favorable OS in R/R AML patients [20]. Also, an interesting previous study discloses that poor risk stratification is correlated with worse OS in R/R AML patients [23]. Another study applies CLAG-M salvage regimen in 118 R/R AML patients from multicenter, and reveals that poor risk stratification is associated with shorter OS, which is the only predictive factor for reduced DFS [24]. These data indicate that higher risk stratification correlates with poor prognosis of R/R AML, as well as elderly age and larger number of prior induction therapies. In line with these studies, we found that CR at first induction was associated with favorable CR, while age ≥ 60 years, higher risk stratification, BM blast ≥ 42.7% and second or higher salvage therapy were associated with unfavorable CR. Moreover, age ≥ 60 years, secondary disease, poor risk stratification, BM blast ≥ 42.1% and second or higher salvage therapy were correlated with shorter OS. When further analyzed in multivariate models that age ≥ 60 years, higher risk stratification, BM blast ≥ 42.7%, secondary disease and second or higher salvage therapy in our study were verified to be independent factors affecting poor prognosis. It was different with aforementioned research that CLAG was not a predictive factor for favorable OS in our study, and this fact might due to: (1) age of patients in our study was younger than theirs; (2) patients in our study received standard dose of FLAG treatments, while part of patients in Hyunkyung Park et al. study receive modified fludarabine-containing therapy which dose is decreased, and this may lead to the different outcomes.

To further investigate difference of outcomes between CLAG and FLAG, analysis of outcomes in subgroups is required. The retrospective study comparing CLAG and FLAG performed by Hyunkyung Park et al. reveals that CLAG achieves better OS and RFS in patients with CR at first induction, de novo AML and relapsed AML without poor risk cytogenetics compared to FLAG, while FLAG obtains preferable OS in patients with secondary AML [20]. In our study, CLAG presented a numerically higher CR in patients with second or higher salvage therapy, numerically prolonged OS in patients with relapsed AML or poor risk stratification, and significant longer OS in patients with CR at first induction. Partially in line with previous research, we also disclosed that CLAG might attain more satisfactory OS in patients with CR at induction and relapsed AML, but we found no difference of OS between CLAG and FLAG in patients with de novo or secondary AML, and this might result from: (1) patients in previous study are older than ours; (2) in our study, 5/55 patients in CLAG group were combined with mitoxantrone treatment, and 6/48 patients in FLAG group were combined with previous idarubicin treatment. While the numbers with mitoxantrone in CLAG and idarubicins in FLAG were 52.3 and 60%, respectively, in their research and, thus, difference of treatments between previous and our study may lead to different outcomes in subgroup analysis.

This study also had limitations: (1) sample size was relatively small; (2) our median follow-up time of 9 months was short, and the long-term survivals by CLAG and FLAG were not evaluated; (3) this was a prospective cohort study and the results would be affected by patients’ characteristics and, thus, a following randomized control study was necessary.

In conclusion, CLAG was equally effective to FLAG induction chemotherapy in total R/R AML patients, while CLAG seemed to be a better option than FLAG in patients with relapsed disease, poor risk stratification, CR at first induction or second or higher salvage therapies.

## Electronic supplementary material

Below is the link to the electronic supplementary material.

Supplementary material 1 (PDF 426 kb)

## References

[1] Dohner H, Weisdorf DJ, Bloomfield CD (2015). Acute myeloid leukemia. N Engl J Med.

[2] Beutler E (1992). Cladribine (2-chlorodeoxyadenosine). Lancet.

[3] Breems DA, Van Putten WL, Huijgens PC, Ossenkoppele GJ, Verhoef GE, Verdonck LF (2005). Prognostic index for adult patients with acute myeloid leukemia in first relapse. J Clin Oncol.

[4] Abutalib S, Tallman MS, Estey EH, Faderl SH, Kantarjian HM (2008). Relapsed and refractory acute myeloid leukemia. Acute leukemias.

[5] Martin MG, Augustin KM, Uy GL, Welch JS, Hladnik L, Goyal S (2009). Salvage therapy for acute myeloid leukemia with fludarabine, cytarabine, and idarubicin with or without gemtuzumab ozogamicin and with concurrent or sequential G-CSF. Am J Hematol.

[6] Kim H, Lee JH, Joo YD, Bae SH, Lee JH, Kim DY (2016). A prospective, multicenter phase II study of continuous infusion of FLAG for patients older than 60 yr with resistant acute myeloid leukemia: a comparison with intensive younger patients’ trial. Eur J Haematol.

[7] Lee SR, Yang DH, Ahn JS, Kim YK, Lee JJ, Choi YJ (2009). The clinical outcome of FLAG chemotherapy without idarubicin in patients with relapsed or refractory acute myeloid leukemia. J Korean Med Sci.

[8] Virchis A, Koh M, Rankin P, Mehta A, Potter M, Hoffbrand AV (2004). Fludarabine, cytosine arabinoside, granulocyte-colony stimulating factor with or without idarubicin in the treatment of high risk acute leukaemia or myelodysplastic syndromes. Br J Haematol.

[9] Ramos NR, Mo CC, Karp JE, Hourigan CS (2015). Current approaches in the treatment of relapsed and refractory acute myeloid leukemia. J Clin Med..

[10] Robak T (2003). Purine nucleoside analogues in the treatment of myleoid leukemias. Leuk Lymphoma.

[11] Van den Neste E, Cardoen S, Offner F, Bontemps F (2005). Old and new insights into the mechanisms of action of two nucleoside analogs active in lymphoid malignancies: fludarabine and cladribine (review). Int J Oncol.

[12] Spurgeon S, Yu M, Phillips JD, Epner EM (2009). Cladribine: not just another purine analogue?. Expert Opin Investig Drugs.

[13] Vahdat L, Wong ET, Wile MJ, Rosenblum M, Foley KM, Warrell RP (1994). Therapeutic and neurotoxic effects of 2-chlorodeoxyadenosine in adults with acute myeloid leukemia. Blood.

[14] Wrzesien-Kus A, Robak T, Lech-Maranda E, Wierzbowska A, Dmoszynska A, Kowal M (2003). A multicenter, open, non-comparative, phase II study of the combination of cladribine (2-chlorodeoxyadenosine), cytarabine, and G-CSF as induction therapy in refractory acute myeloid leukemia—a report of the Polish Adult Leukemia Group (PALG). Eur J Haematol.

[15] Duan MH, Zhang Y, Zhang M, Han X, Zhang Y, Yang C (2016). Efficacy and safety analysis of the combination of cladribine, cytarabine, granulocyte colonystimulating factor (CLAG) regime in patients with refractory or relapsed acute myeloid leukemia. Zhonghua Xue Ye Xue Za Zhi..

[16] NCCN Clinical Practice Guidelines in oncology: acute myeloid leukemia (Version 1. 2016).

[17] Touw IP, van de Geijn GJ (2007). Granulocyte colony-stimulating factor and its receptor in normal myeloid cell development, leukemia and related blood cell disorders. Front Biosci..

[18] Liongue C, Ward AC (2014). Granulocyte colony-stimulating factor receptor mutations in myeloid malignancy. Front Oncol..

[19] Price SL, Lancet JE, George TJ, Wetzstein GA, List AF, Ho VQ (2011). Salvage chemotherapy regimens for acute myeloid leukemia: is one better? Efficacy comparison between CLAG and MEC regimens. Leuk Res.

[20] Park H, Youk J, Kim I, Yoon SS, Park S, Lee JO (2016). Comparison of cladribine- and fludarabine-based induction chemotherapy in relapsed or refractory acute myeloid leukaemia. Ann Hematol.

[21] Becker PS, Kantarjian HM, Appelbaum FR, Storer B, Pierce S, Shan J (2013). Retrospective comparison of clofarabine versus fludarabine in combination with high-dose cytarabine with or without granulocyte colony-stimulating factor as salvage therapies for acute myeloid leukemia. Haematologica.

[22] Holowiecki J, Grosicki S, Giebel S, Robak T, Kyrcz-Krzemien S, Kuliczkowski K (2012). Cladribine, but not fludarabine, added to daunorubicin and cytarabine during induction prolongs survival of patients with acute myeloid leukemia: a multicenter, randomized phase III study. J Clin Oncol.

[23] Kim H, Seol YM, Song MK, Choi YJ, Shin HJ, Park SH (2016). Evaluation of prognostic factors in patients with relapsed AML: clonal evolution versus residual disease. Blood Res..

[24] Wierzbowska A, Robak T, Pluta A, Wawrzyniak E, Cebula B, Holowiecki J (2008). Cladribine combined with high doses of arabinoside cytosine, mitoxantrone, and G-CSF (CLAG-M) is a highly effective salvage regimen in patients with refractory and relapsed acute myeloid leukemia of the poor risk: a final report of the Polish Adult Leukemia Group. Eur J Haematol.

