# Supplementary material for: Comparison of clinical remission and survival between CLAG and FLAG induction chemotherapy in patients with refractory or relapsed acute myeloid leukemia: a prospective cohort study
Source: Clin Transl Oncol. 2017 Nov 27;20(7):870–80. doi: 10.1007/s12094-017-1798-8 (PMC5996008; doi:10.1007/s12094-017-1798-8)
Supplement: Supplementary file 1 — Supplementary material 1 (PDF 426 kb) [file 12094_2017_1798_MOESM1_ESM.pdf]

Comparison of clinical remission and survival between CLAG and FLAG induction chemotherapy in patients with refractory or relapsed acute myeloid leukemia: a prospective cohort study

Running title: CLAG versus FLAG in treating R/R AML patients

Ying Bao[1], Juan Zhao[2], Zhang-Zhi Li[3*]

[1]Department of Hematology, Xiangyang NO. 1 People's Hospital, Affiliated to Hubei University of Medicine, Xiangyang, China;

[2]Department of Hematology, The Central Hospital of Wuhan, Tongji Medical College, Huazhong University of Science and Technology, Wuhan, China;

[3]Department of Hematology, Taihe Hospital Affiliated to Hubei University of Medicine, Shiyan, China.

Ying Bao and Juan Zhao contributed equally to this work.

***Correspondence:** Zhangzhi Li,

Department of Hematology, Taihe Hospital Affiliated to Hubei University of Medicine, 32 Renmin South Road, Shiyan 442099, China.

Tel: +86-719-8801443;

Fax: +86-719-8801443;

Email: lizhangzhi07@outlook.com

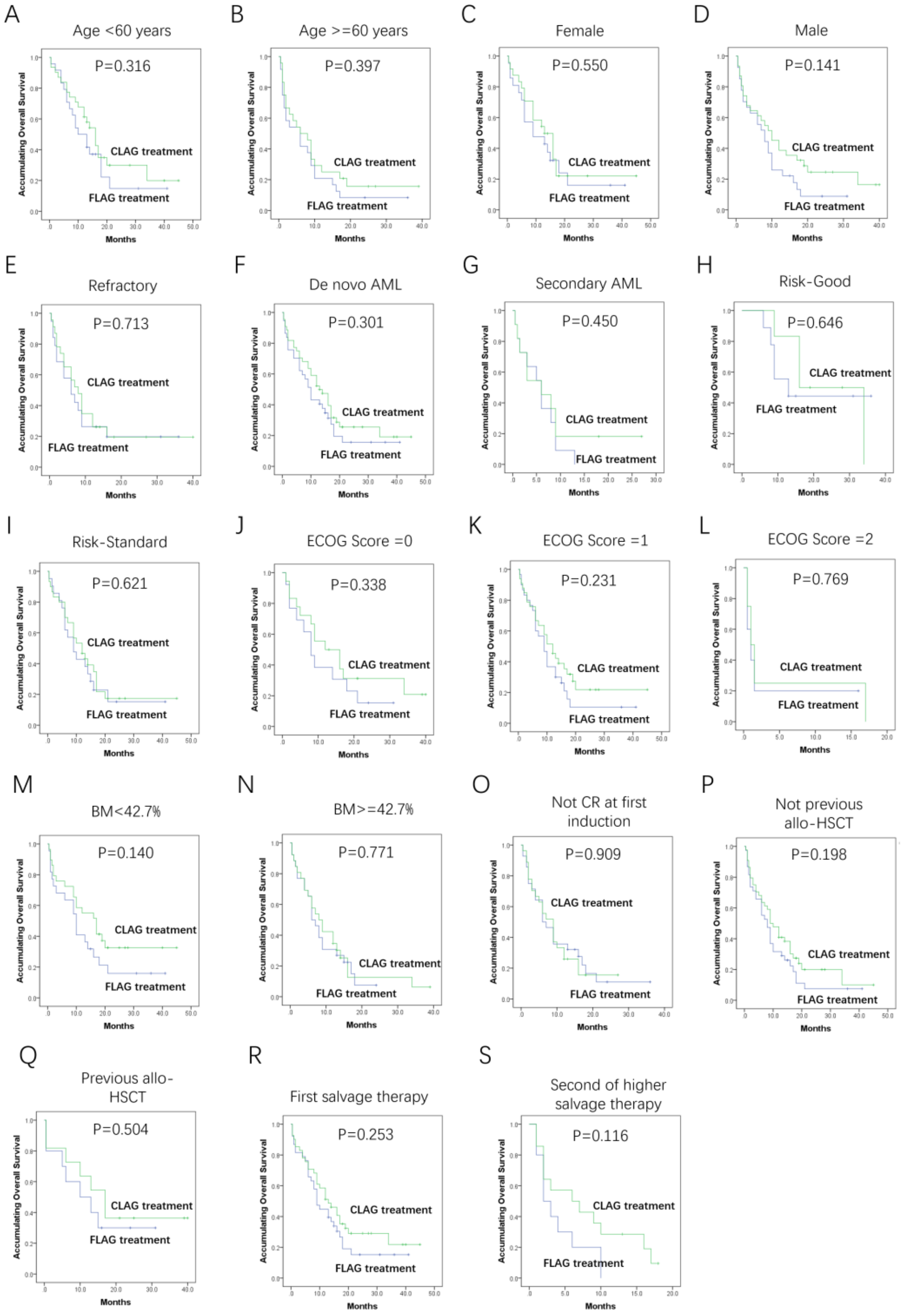

Supplemental Figure S1 Accumulating OS between CLAG and FLAG treatments in subgroups divided by baseline characteristics (Data with *p* value >= 0.1). No difference of OS between CLAG and FLAG in these subgroups was discovered (A, B, C, D, E, F, G, H, I, J, K, L, M, N, O, P, Q, R, S). Comparison of OS between groups was determined by K-M curves and log-rank test. *p*<0.05 was considered significant.
